# Bilateral Sector Macular Dystrophy Associated with *PRPH2* Variant c.623G>A (p.Gly208Asp)

**DOI:** 10.3390/jcm14144893

**Published:** 2025-07-10

**Authors:** Simone Kellner, Silke Weinitz, Ghazaleh Farmand, Heidi Stöhr, Bernhard H. F. Weber, Ulrich Kellner

**Affiliations:** 1 Rare Retinal Disease Center, AugenZentrum Siegburg, MVZ Augenärztliches Diagnostik-und Therapiezentrum Siegburg GmbH, 53721 Siegburg, Germany; s.kellner@osg.de (S.K.); s.weinitz@osg.de (S.W.); g.farmand@osg.de (G.F.); 2 RetinaScience, 53192 Bonn, Germany; 3 Institute of Human Genetics, University of Regensburg, 93053 Regensburg, Germany; heidi.stoehr@klinik.uni-regensburg.de (H.S.); bernhard.weber@klinik.uni-regensburg.de (B.H.F.W.); 4 Institute of Clinical Human Genetics, University Hospital Regensburg, 93503 Regensburg, Germany

**Keywords:** *PRPH2*, macular dystrophy, retinal imaging, phenotype

## Abstract

**Objective**: The clinical presentation of inherited retinal dystrophies associated with pathogenic variants in *PRPH2* is highly variable. Here we present bilateral sector macular dystrophy as a novel clinical phenotype. **Methods and analysis**: Ophthalmologic examination, detailed retinal imaging with optical coherence tomography (OCT), OCT-angiography, fundus and near-infrared autofluorescence and molecular genetic testing were performed on a 30-year-old female. **Results**: The patient reported the onset of subjective visual disturbances 4.5 months prior to our first examination. Clinical examination and retinal imaging revealed bilateral sharply demarcated paracentral lesions in the temporal lower macula and otherwise normal retinal findings. Patient history revealed no medication or other possible causes for these unusual retinal lesions. Molecular genetic testing revealed a heterozygous c.623G>A variation (p.(Gly208Asp)) in the *PRPH2* gene. **Conclusions**: Bilateral sectoral macular dystrophy has not been reported previously in any inherited retinal dystrophy. This feature adds to the wide spectrum of *PRPH2*-associated clinical presentations.

## 1. Introduction

Inherited retinal dystrophies (IRD) associated with pathogenic or likely pathogenic variants in the *PRPH2* gene are known to present with a broad clinical spectrum of retinal manifestations even within the same family [1,2,3,4]. Recently, five to seven phenotypes have been defined based on clinical findings as well as fundus autofluorescence [3,4,5], although different terminology has been used for some phenotypes in these studies. Most lesions predominantly affect the macular area: central areolar choroidal dystrophy, butterfly-shaped pattern dystrophy, adult-onset vitelliform macular dystrophy, and macular dystrophy. Three types affect the retina in general: pseudo-Stargardt macular dystrophy (multifocal pattern dystrophy or multifocal pattern dystrophy simulating fundus flavimaculatus), retinitis pigmentosa and extensive chorioretinal atrophy similar to choroideremia. In other reports, cone-rod dystrophy has been described, which might be included as a late stage of pseudo-Stargardt macular dystrophy. Moreover, asymptomatic carriers with normal fundus and normal retinal function on electroretinography have been identified during family examinations [1,3,4,5,6].

A total of 296 unique pathogenic/likely pathogenic variants in the *PRPH2* gene have so far been listed on the *PRPH2* gene homepage of the LOVD database [7]. Of these, only a small proportion show a reasonable genotype-phenotype correlation, whereby the associated phenotypes largely belong to the group of autosomal dominant retinitis pigmentosa [2]. Thirteen missense variants are associated with central areolar choroidal dystrophy [3]. For most of the other variants, the manifesting phenotypic variability is high.

Here we describe a female patient with a unique presentation of macular involvement with bilateral sector macular dystrophy carrying a heterozygous c.623G>A variant in the *PRPH2* gene, resulting in a glycine-to-aspartic acid substitution at position 208 in the peripherin-2 protein. The unique macular lesions, to the best of our knowledge, have neither been reported in patients with causative *PRPH2* variants nor in any other IRD.

## 2. Materials and Methods

### 2.1. Clinical Diagnosis

The patient underwent best corrected visual acuity testing, automated visual field testing (HFA3, type 840, Carl Zeiss Meditec, Oberkochen, Germany) and anterior and posterior segment biomicroscopy. Fundus photography was performed with wide-angle imaging (Zeiss Clarus 700, Carl Zeiss Meditec, Oberkochen, Germany). Retinal imaging, including multicolor spectral reflectance imaging, fundus autofluorescence (FAF), near-infrared autofluorescence (NIA), spectral-domain optical coherence tomography (OCT) and OCT-angiography (OCTA), was performed as described previously [8,9]. All images were obtained after medical dilatation of the pupil (phenylephrine 2.5% and tropicamide 1% achieving a minimal diameter of 5 mm) by trained retinal imaging specialists. FAF and NIA were obtained with a confocal scanning laser ophthalmoscope (Heidelberg Retina Angiograph 2, Heidelberg Engineering, Heidelberg, Germany) using 30° (M-FAF, M-NIA) and 55° lenses (W-FAF, W-NIA). Multicolor spectral reflectance images and OCT were obtained with a spectral domain OCT (Spectralis OCT, Heidelberg Engineering, Heidelberg, Germany). A standard volume scan macular OCT (M-OCT) was recorded using 49 B-scans with a distance between B-scans of 129 μm in a 20 × 20-degree field (6.2 mm × 6.2 mm) using ART mode with 16 images averaged. A wide-field (W-OCT) was recorded using 31 B-scans with a distance between B-scans of 245 μm in a 55 × 25-degree field (16.1 mm × 7.3 mm) using ART mode with 16 images averaged. OCT-angiography (OCT-A) was acquired with a Spectralis OCT2 (Heidelberg Engineering GmbH, Heidelberg, Germany; OCT Camera Version 1.6.5.0, Acquisition Software Version 6.7.21.0) in an examination field size of 3.2 × 3.2 mm.

### 2.2. Molecular Genetic Testing

DNA was extracted from peripheral blood according to established methods. DNA diagnostics was performed via next-generation sequencing (NGS)-based custom-designed multigene panel testing comprising 289 genes previously linked to inherited retinal dystrophies [10]. Classification of genetic variants was achieved according to joint consensus recommendations of the American College of Medical Genetics and Genomics (ACMG) and the Association for Molecular Pathology (AMP) [11].

## 3. Results

The 30-year-old female patient (#4890_F554) presented with subjective visual disturbances, including glare, problems with focusing and a different image size in both eyes. Symptoms were noted after returning to work following vacancies. Initially, a slight dizziness and mild headache were noted when looking with both eyes; occlusion of either eye removed these symptoms. No specific health problems at onset or in the weeks prior to first signs of visual impairment, e.g., a cold, as well as no other generalized symptoms or intake of medication, were reported at detailed questioning. Initially, the ocular problems were presumed to be of neurologic origin; however, a subsequent detailed neurologic work-up (clinical neurologic examination, cranial computer tomography, magnetic resonance imaging, cerebrospinal fluid examination and blood analysis for inflammation markers) remained unremarkable. The first ophthalmic examination was performed elsewhere 6 weeks after the initial visual symptoms, and bilateral paramacular lesions were observed.

The first examination in our department was performed 4.5 months after the onset of symptoms. In between, the subjective complaints remained stable, with no increase or decrease over time.

**Figure 1:**
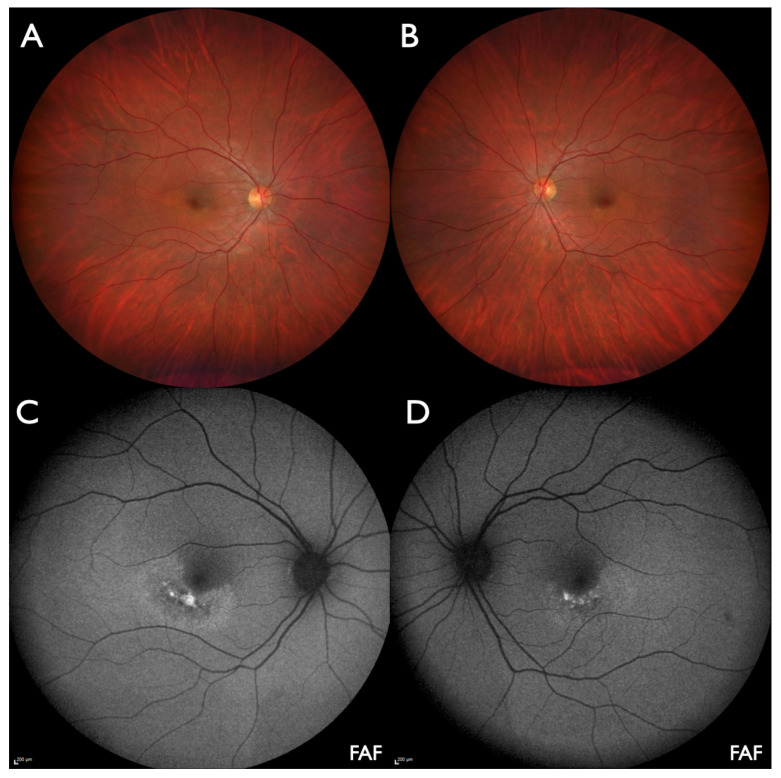
Index patient (#4890_F554): (**A**,**B**) Wide-field color images show slightly curved paracentral lesions below and temporal to the fovea on the right (**A**) and left eye (**B**). (**C**,**D**) Wide-field fundus autofluorescence (W-FAF) more clearly shows the sharply demarcated areas with fleck-like increased and reduced FAF intensity on the right (**C**) and left eye (**D**).

**Figure 2:**
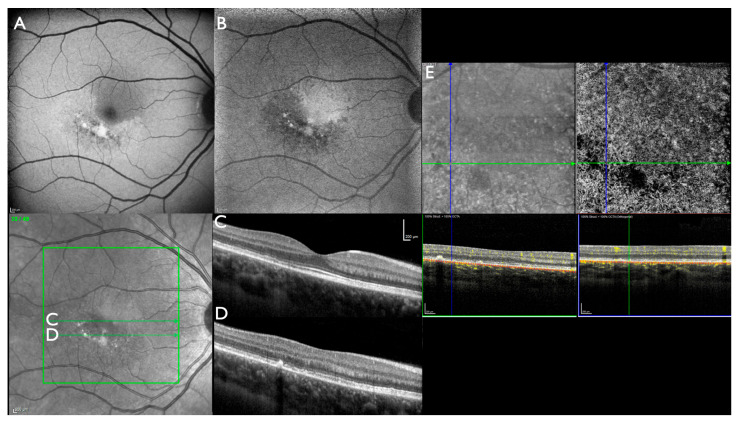
Index patient (#4890_F554), right eye: (**A**) Macular fundus autofluorescence (M-FAF) shows the sharply demarcated area with fleck-like increased and reduced FAF intensity. (**B**) Macular near-infrared autofluorescence (M-NIA) shows the sharply demarcated area with reduced NIA intensity and few fleck-like spots of increased and markedly reduced NIA intensity. (**C**) Optical coherence tomography (OCT) macular foveal scan: mild irregularities temporal of the fovea from the ellipsoid to the interdigitation zone. (**D**) OCT macular lower scan with moderate irregularities from the external limiting membrane to the retinal pigment epithelium. (**E**) OCT-angiography: reduced choriocapillaris flow within the lesion.

Visual acuity was 20/20 bilaterally with emmetropic refraction. Automated visual fields were normal. Biomicroscopy of the anterior segment and the ocular media was normal. Ophthalmoscopy revealed normal optic disks, retinal vessels, and retinal periphery in both eyes. At the posterior pole, slightly curved paracentral lesions below and temporal to the fovea were observed (Figure 1A,B). Both lesions were better visualized using FAF and NIA (Figure 1C,D and Figure 2A,B). OCT showed loss of outer retinal structures and fleck-like subretinal deposits within the lesions but was normal outside of the damaged area (Figure 2C,D). Central choroidal thickness was in the upper normal range (OD: 280 µm, OS: 320 µm), and there was no difference between affected and unaffected areas throughout the volume scan. OCTA detected reduced vascular density in the deep capillary plexus within the affected area but was normal for the DCP and all other layers outside the lesions (Figure 2E). Subjectively, prism glasses improved near vision but not far vision.

Initially, and based on the subjectively noted onset, a non-hereditary origin of the disorder was suspected, e.g., acute macular neuroretinopathy, paracentral acute middle maculopathy or multiple evanescent white dot syndrome. However, the bilateral similar retinal lesions, the missing correlation of retinal imaging findings to possible acquired disorders, and the absence of any indicators for other disorders in the patient’s history led us to initiate molecular genetic testing.

Multigene panel diagnostics identified a heterozygous c.623G>A variant in exon 2 of the *PRPH2* gene (transcript reference sequence: NM_000322.5), which is expected to result in a replacement of the highly conserved glycine at codon 208 with aspartic acid, p.(Gly208Asp). This genetic variant has rarely been found in the general population (gnomAD v4.1: 139/1.614.058 alleles). The missense variant c.623G>A/p.(Gly208Asp) in the *PRPH2* gene was classified as likely pathogenic based on ACMG criteria (PM2, PP3, PS4-moderate, PP1).

This variant has been observed in the heterozygous state with moderate frequency in less than 40 affected patients with autosomal-dominant retinal disease, most patients from Spain [12,13,14,15,16], but also from Brazil [17], France [18], Germany [19,20,21], Italy [5,22], the Netherlands [2,23], Norway [24], the United Kingdom [25,26] and the USA [1,6,27,28,29]. In some cases it was found to segregate with disease in related individuals (e.g., [20,28]). When reported, clinical presentation ranged between unaffected older family members carrying the c.623G>A variation, Stargardt-like macular dystrophy or pattern dystrophy and severe retinitis pigmentosa.

**Figure 3:**
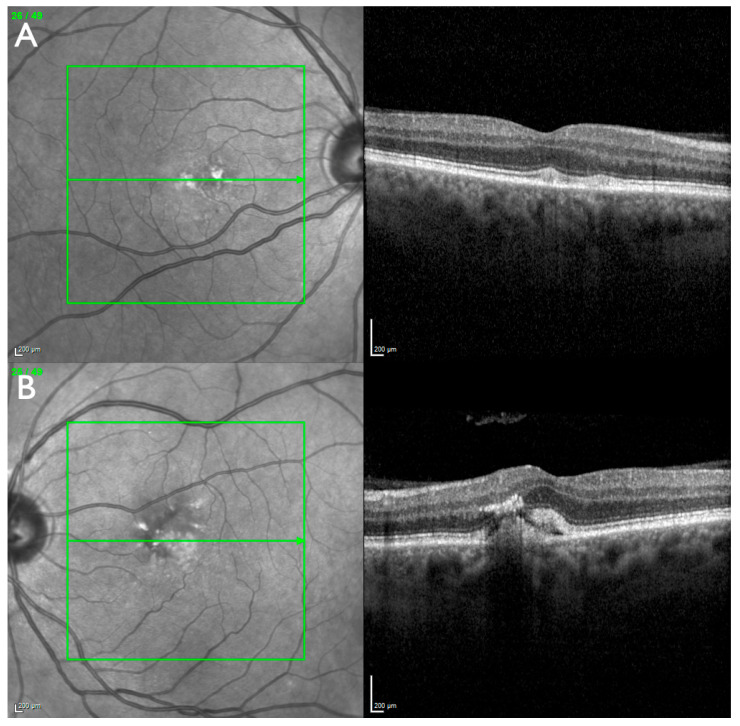
Father of index patient (#6096_F554): Macular optical coherence tomography (M-OCT). (**A**) On the right eye, subfoveal and nasal to the fovea, two lesions with material of increased reflectance are present between the ellipsoid zone and the interdigitation zone. (**B**) On the left eye, a thickened retina with subfoveal disorganization of the outer retinal layers, subretinal hyperreflective material, subretinal fluid and hyperreflective intraretinal foci is present.

The patient has no children. Her half-sister was reported to be clinically unaffected but, to our knowledge, was not examined ophthalmologically. The patient’s father (#6096_F554) presented in our clinic once at the age of 68 years. He was undergoing intravitreal anti-VEGF therapy on the left eye for presumed neovascular age-related macular degeneration in another clinic. Subretinal lesions under and adjacent to the fovea were present in both eyes, and foveal subretinal fluid and intraretinal hyperreflective lesions were present in the left eye (Figure 3). No atrophic macular lesions or other peripheral retinal abnormalities were observed. The absence of drusen and the lesions seen on OCT could be interpreted as a form of adult-onset vitelliform macular dystrophy with an associated macular neovascularization in the left eye. The patient’s mother has a history of visual problems but denied ophthalmological examination. Neither parent consented to molecular genetic testing.

**Figure 4:**
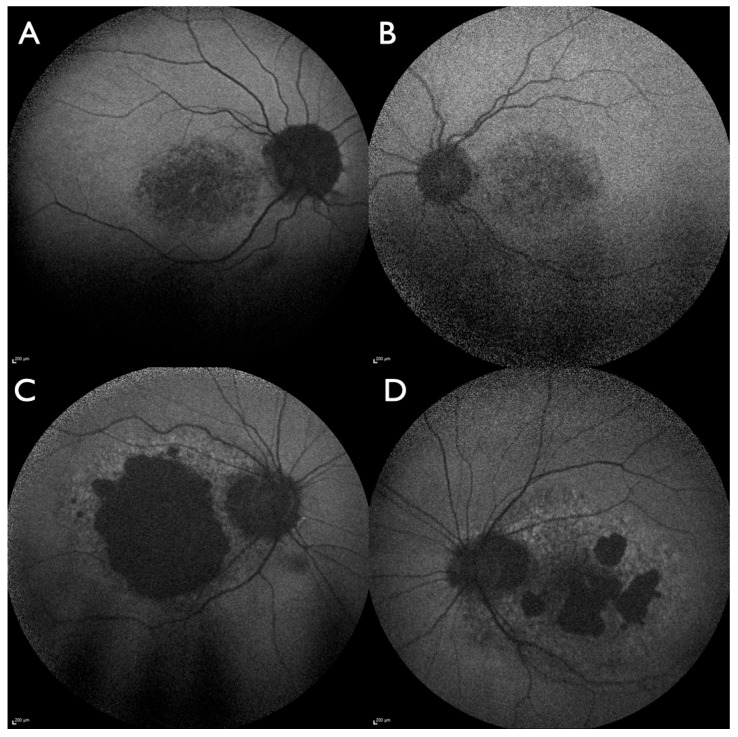
Two unrelated patients with the same (p.(Gly208Asp)) genotype and a differently progressed central areolar choroidal dystrophy phenotype at nearly the same age. (**A**,**B**) Patient (#4264, 68 years of age) wide-field fundus autofluorescence (W-FAF). The macular area is affected with peripapillary markedly reduced FAF intensity in the right eye (**A**) but not in the left eye (**B**) and moderately reduced FAF intensity at the posterior pole in both eyes. (**C**,**D**) Patient (#4172, 69 years of age) W-FAF: Predominantly the macular area is affected with peripapillary markedly reduced FAF intensity in both eyes. On the right eye (**C**), a large area with markedly reduced FAF intensity is surrounded by fleck-like irregular FAF intensity. In the left eye (**D**), smaller areas with markedly reduced FAF intensity are surrounded by fleck-like irregular FAF intensity.

Our clinical database includes 71 molecularly confirmed IRD patients with causative *PRPH2* variants. In addition to the index patient described here, two other unrelated patients presented with the (p.(Gly208Asp)) genotype: one female (#4264; 68 years of age at first examination, onset noted at 58 years of age, no other family members affected) and one male (#4172; 69 years of age at first examination, onset noted at 45 years of age, mother affected). Both presented with severe macular involvement diagnosed as central areolar choroidal dystrophy (Figure 4). Patient #4264 showed preserved small foveal areas on OCT and pericentrally reduced but preserved FAF, corresponding with pericentral scotoma and a visual acuity of 20/30 in both eyes. Patient #4172 showed a large area of RPE and photoreceptor atrophy involving the fovea on the right eye and pericentral atrophic areas with a preserved small foveal area in the left eye. Visual acuity was 20/500 on the right eye and 20/40 in the left eye.

## 4. Discussion

To the best of our knowledge, bilateral paracentral lesions in the lower temporal region of the macula as identified in our patient have not been described before in association with variants in *PRPH2* or in any other inherited or acquired retinal disease.

The possible origin of novel phenotypes has to be carefully evaluated. The patient reported noting symptoms when returning to work following vacancy, which may indicate a sudden onset subsequent to previous activities. Travel during vacancies was limited to a Spanish island, which makes an acquired infectious disorder unlikely. The intake of medicine or drugs, e.g., poppers, was denied. In the month prior to the visual symptoms, no cold, which might precede acquired disorders like acute macular neuro-retinopathy, paracentral acute middle maculopathy or multiple evanescent white dot syndrome, was reported, and the clinical and imaging findings excluded those differential diagnoses. Another possible differential diagnosis is pachychoroid pigment epitheliopathy (PPE), which can be difficult to distinguish from macular dystrophies [30]. However, PPE usually presents with few or multiple smaller non-confluent lesions and is not symmetrical in both eyes [30,31,32,33]. The defining feature of PPE on OCT is a thick choroid usually located directly beneath the clinically apparent retinal pigment epithelial change [31]. In our patient, though the choroidal thickness was in the upper normal range, no variation in choroidal thickness between normal and affected areas was observed. In conclusion, an acquired origin of the symmetrical bilateral paracentral lesions in our patient is not supported.

Bilateral symmetrical lesions are more likely to occur in IRDs, though the pattern described here has not been observed in a recent series evaluating FAF imaging in more than 3.600 molecularly confirmed IRD patients [34]. The findings in the father with likely adult-onset vitelliform macular dystrophy could support the diagnosis; unfortunately, he declined genetic testing. A similarly located lesion has been observed in one eye of an unrelated patient associated with the p.(Arg172Gln) variant in the *PRPH2* gene; the other eye of that patient presented with a perifoveal lesion similar to central areolar choroidal dystrophy [35]. In the literature and in our own patient database, the c.623G>A variant was found multiple times to be associated with autosomal dominant central areolar choroidal dystrophy (e.g., [3,4,20]) and less frequently with autosomal dominant retinitis pigmentosa (e.g., [18]). Incomplete penetrance has been observed with this variant [6]. In conclusion, bilateral paracentral lesions are one phenotype associated with the c.623G>A variant.

The location of the causative variant affects the D2 loop, which is a hot spot of pathogenic variants in the *PRPH2* gene [2]. Some nearby localized variants (e.g., p.(Pro210Ser), p.(Phe211Leu)) are associated with retinitis pigmentosa, whereas others (e.g., p.(Val209Ile), p.(Val209Phe), p.(Val209Asp)) are associated with variable clinical expression like different forms of macular dystrophy. The D2 loop is a key extracellular domain critical for proper PRPH2 folding and oligomerization and interaction with ROM1. Glycine 208 is a highly conserved, flexible residue, often essential for structural turns or tight packing, while aspartic acid is larger and negatively charged, potentially introducing steric hindrance or electrostatic repulsion that may disrupt local structure or interaction surfaces.

## 5. Conclusions

Bilateral sector macular lesions have not been reported in association with any other retinal disorder previously. This case illustrates that molecular genetic testing is mandatory to identify genetic variants in atypical clinical presentations.

## Data Availability

The data presented in this study are available on request from the corresponding author due to legal restrictions.

## Abbreviations

The following abbreviations are used in this manuscript:

FAF	Multidisciplinary Digital Publishing Institute
IRD	inherited retinal dystrophies
NIA	near-infrared autofluorescence
OCT	optical coherence tomography
OCTA	OCT-angiography
PPE	pachychoroid pigment epitheliopathy
RPE	retinal pigment epithelium
